# Clinical Report on Chronic Pleurisy, Based on an Analysis of Forty-Seven Cases

**Published:** 1853-01

**Authors:** Austin Flint


### BUFFALO MEDICAL JOURNAL

AND

### MONTHLY REVIEW.

**VOL. 8.** JANUARY, 1853. **NO. 8.**

### ORIGINAL COMMUNICATIONS.

ART. I.—*Clinical Report on Chronic Pleurisy, based on an analysis of Forty-seven cases.* By AUSTIN FLINT, M. D.

(Concluded from page 418.)

PLEURISY CONSEQUENT ON PERFORATION OF LUNG.

In four cases pleurisy was developed in consequence of perforation of lung. In two of these cases the perforation occurred in the progress of tuberculous disease, and the pleurisy was, thus, a complication of phthisis. In the remaining two cases circumscribed gangrene of lung preceded the perforation, the latter taking place over the gangrenous part. A succinct account of these cases, severally, may possess some interest.

CASE 1. The patient, a male, was twenty-one years of age. There existed a family predisposition to tubercle, and he had evidently labored under the disease for more than a year. He was not under my observation prior to the perforation. He was able to be up and dressed when the perforation occurred. It took place during a violent fit of coughing, and was signalized by the sudden development of acute pain, and difficulty of breathing. I saw the patient on the third day after the perforation. The pain and dyspnœa was then much diminished. He was unable to lie on the left side, which was the side in which was the perforation. The cough and expectoration were less after the perforation than before. The pulse was from 130 to 140.

The left side was visibly enlarged, and almost immovable in respiration. There was tympanitic resonance on this side except at the upper third posteriorly. Metallic tinkling was distinctly heard over the middle third; three or four tinkling sounds succeeding expiration, and occasionally accompanying inspiration. This sign was uniformly present at repeated acts of listening during the visit. It was absent at a visit on a subsequent day. At the next visit, a few days after the first (the precise number of days does not appear in the record) the left chest was still more largely distended, and the intercostal spaces elevated. He suffered much from dyspnœa.

He died shortly afterward, the precise date not being given. There was no autopsy.

Case 2. The patient was a female, aged about thirty-five. I had examined the chest and determined the presence of tubercle months before the perforation occurred. At that time she was not under my charge. She came under my care in Sept., 1849, and at this time the evidences of perforation were present, and the time of its occurrence could not be ascertained, or, at all events, it does not appear from the notes that it was ascertained. The perforation occurred in the left side. There was tympanitic resonance at the top of the left shoulder and posteriorly at the summit of the chest. Below flatness. A loud cavernous respiration was present. A distinct tinkling sound w heard accompanying both inspiration and expiration, especially the latter; two, three, four and even five tinkling sounds with each act. This sign was not uniformly present. Lying down suddenly frequently occasioned a plashing noise, which had arrested her attention, and had also been frequently heard by her attendants. I was able distinctly to hear it as she lay on her right side and moved the body to and fro. I could not develop it by succussion while she was in a sitting posture.

She was quite feeble, but was accustomed to ride out on every pleasant day.

Pulse 1·20. Respiration accelerated. Some fœtor in the expectoration was occasionally observed, but not in a marked degree.

Death occurred about two months after the case came under my charge. The patient suffered greatly from paroxysm of dyspnœa toward the close of life. There was no autopsy.

CASE 3. The patient, a male, was forty-five years of age. He had suffered from epilepsy for two years, which had considerably impaired the mental faculties. He had long been accustomed to use ardent spirits freely, and sometimes to excess.

I saw the patient, first, on the 28th Jan., 1852, in consultation. He had the day previous had a severe epileptic paroxysm. Prior to this date he had not had an attack for some time. At the time of my visit he appeared much prostrated, and had some cough with expectoration. On physical exploration I found only some bronchial râles, without any of the signs of solidification, and I supposed he was simply laboring under an attack of bronchitis.

I did not again visit him until the 5th of February. He then had a very fœtid expectoration, of a gangrenous odor; and there were evidences of solidification at the inferior part of the chest on the right side anteriorly and posteriorly.

I saw him next on the 8th instant. The fœtid expectoration had ceased. He now had little or no expectoration. His breath was occasionally fœtid. He was greatly prostrated. The respirations were frequent and labored. Pulse frequent and tolerably developed. Some delirium. He had had a chill, and experienced acute pain in the right chest. He took food without reluctance and with some relish. On examination of the chest I found the physical evidences of perforation and pneumo-hydro-thorax as follows: tympanitic resonance over the upper and middle thirds of right chest anteriorly. Metallic tinkling at the inferior third just below the level of the nipple. The respiration over the middle third was blowing and low in pitch. At the upper third it was bronchial, i. e. tubular and high in pitch. Posteriorly at the upper third the respiration was bronchial. Over the middle third, posteriorly, it was blowing, feeble and low in pitch.

Death occurred on the night of the 10th of February, and the chest was examined, *post mortem*, on the 13th, by Prof. John C. Dalton, jr., who furnished the following report of the morbid appearances:*

"Autopsy of Mr. S——, February 13th, 1852. Oo npening the cavity of the right pleura, a considerable quantity of rathe· fœtid gas escaped, and on raising the sternum the same cavity was found to contain about two pints of a dingy, yellowish-gray purulent fluid. The pleural surface at the anterior part of the chest was covered by a thin coating of recent lymph.

* This is one of the cases given in the Appendix to Prize Essay on Variations in Pitch, etc.

"The right lung, reduced to about one-fifth of its natural size, was compressed against the spine and the posterior wall of the chest. Before removing the fluid from the pleural cavity, a pipe was inserted into the trachea, and on inflating the lungs the air escaped freely in large bubbles, from a point situated toward the posterior part of the right lung, about the junction of its upper and middle thirds. The right lung, removed from the chest, was found to be completely carnified by pressure, except in those parts occupied by disease. No air-cells were visible anywhere, and the whole lung was destitute of crepitation. The lobes were adherent to each other by recent lymph. The upper third of the lower lobe was occupied by gangrene, which had reached an advanced stage, the pulmonary tissue forming a soft, shreddy, disintegrated mass of a fœtid odor, and a dirty, grayish color, and infiltrated with purulent fluid. A gangrenous cavity of considerable size, had apparently existed at this spot before the compression of the lung. The opening by which it had communicated with the pleural cavity was about the size of a goose-quill, and situated posteriorly at the very uttermost portion of the lower lobe. The limits of the grangrenous portion of the lung, were well defined, but only a very little inflammation existed in its neighborhood, the solidification of the pulmonary tissue, except just outside the limits of this gangrenous cavity, being entirely of a passive character, and due to its compression by the pleuritic effusion. The left lung and pleura were healthy. There was no tubercle anywhere."

Case 4. The patient was an Irish laborer, aged about thirty. He had complained of pain in the left side for two months, and had received medical treatment at the hands of Dr. Ring, of this city. The pain was not very severe, nor accompanied by sufficient embarrassment of respiration to prevent him from laboring. He was a hard-working man, not intemperate, so far as Dr. R. could learn, but lived in dirt and destitution, two families, each consisting of several members, occupying one small room, in an insalubrious part of the city.

About a week before his death the pain was suddenly increased; respiration labored; moderate febrile movement became developed, and he had a hard spasmodic cough. He was bled by Dr. R., took cathartics, with nauseating doses of ipecacuanha and antimony. The symptoms were not relieved. I saw him, with Dr. R., two days before his death, but owing to the filth of the place, and personal indisposition, the physical examination was very hurried. I found flatness on percussion over the lower part of the left chest, and, in front, absence of respiratory sound. Posteriorly I thought I

recognized a faint crepitation. The pulse was 120 and tolerably developed. He complained of pain in the left side on respiration. The chest on the left side was tender on percussion. He had had considerable expectoration, which Dr. Ring had repeatedly inspected with care. It had not been fœtid.

On the day before his death he was transferred to the Hospital of the Sisters of Charity. Dr. Geo. N. Burwell, the attending physician, at that time on service, saw him once. He was then suffering from severe pain in the left side, with labored respiration. Dr. B. found flatness on percussion at the inferior part of the left chest, tympanitic resonance at the superior part, and well-marked metallic tinkling. The heart was displaced, so that its motions were visible to the right of the sternum. Exaggerated respiration existed on the right side. Fœtor of the expectoration was not observed while he was in the hospital. Death occurred, by apnœa.

At the autopsy, fourteen hours after death, at which I was present by invitation of Dr. B., the following morbid appearances were revealed:—The chest being opened, a quantity of fœtid gas escaped. The left pleural cavity was filled with dirty ditch-water-like fluid, with shreds of fibrin. The fœtor was strong and characteristic. The quantity of liquid was estimated to be three or four quarts. Before removing all the liquid, the trachea was opened, and on introducing a blow-pipe, the perforation was demonstrated by large bubbles at the lower part of the chest. On removing the liquid, the pleura was found to be covered with layers of semi-organized fibrin. The pleurisy had evidently been of longer duration than the illness of the week immediately preceding his death, i. e. from the date of his giving up work and taking to the bed. It had doubtless existed while he continued at work, complaining of pain in the side. The lung on the left side was compressed to the back part of the chest, but extended to the lower part, having become attached at its inferior extremity to the diaphragm. It was free from tubercle. It was not solidified except by compression. At the lower part of the inferior lobe a perforation existed large enough to receive the end of the little finger. This opened into a gangrenous cavity of the size of a small English walnut. This cavity presented, all sides, a gangrenous layer about half an inch in thickness. The gangrene was distinctly circumscribed, and no solidification in its neighborhood. On closing the perforation the lung on this side was readily inflated.

In the case last given, the occurrence of gangrene with perforation as a complication of Chronic Pleurisy is an interesting fact. The absence of all fœtor in the expectoration in this case is worthy of note.

It will be perceived that in each of the four cases the existence of perforation was evidenced by the pathognomonic physical sign — metallic tinkling. So far as these few cases go, they show the value which this sign derives from its constancy, being one of the most characteristic of all the physical signs when present.

The cases illustrate the speedily fatal termination, by apnœa, which generally follows the occurrence of perforation; and, also, the fact that occasionally life may be prolonged under these circumstances for several weeks, and even months.

### CASES OF EMPYEMA.

The liquid contents of the pleural cavity were ascertained to be purulent in three cases only. In one of these cases a collection of pus took place beneath the skin communicating with the cavity of the chest, which was opened, and the contents discharged. In the other cases death occurred without any operation. An abstract of the history in each of the cases will not occupy much space.

Case 1. The patient was a male, aged thirty, a cabinet-maker, of intemperate habits. The case came under my observation in January 12, 1841. He was attended at that time by Dr. Samo, of this city. He was ill in a refectory, without conveniences or comforts. At the time I visited him under these circumstances the following were the symptoms: — Face ghastly, and expression anxious. Respiration labored. Anorexia. Incessant hacking cough. Pulse at morning of normal frequency, at evening accelerated. Voice hollow. Great restlessness, frequently getting out of bed. Shortly afterward the pulse became very frequent and intermitting; the lower extremities cold; lividity at roots of nails, and a fatal termination was daily expected. He retained muscular strength to a surprising degree, getting out of bed, and insisting upon sitting up, while the foregoing symptoms were present.

After a few days, improvement took place. The chest, which had not before been examined with much care, was now found to be enlarged on the left side, and universal flatness on percussion, with absence of respiratory sound. He now had good appetite; obtained quiet sleep; respiration became less labored. He had occasionally pretty severe pain in the left chest. Some œdema of feet. Pain in feet, and some inability to control motions of lower extremities. The action of the heart occasioned a thrill or jarring motion over the whole chest, no point of impulse being perceptible. The case ended fatally February 26, 1841.

The left pleural cavity was found to contain several quarts of laudable, inodorous pus. Pleural surfaces covered with layers of fibrin. Lung compressed to a mass of the breadth and thickness of the hand. No tubercle. Some adhesions in the right side; otherwise no morbid appearances. Moderate hypertrophy of the left ventricle of heart existed. Aortic and left auriculo-ventricular valves thickened.

Case 2. The patient in this case was a female child, aged six years, delicate, but usually had good health. She was attacked December 14, 1845, with vomiting, accompanied by febrile movement. Stupor followed, leading to the supposition of congestion or effusion within the head. She emerged from this state, and appeared to be improving, when, December 22, there was a change for the worse. Cough, which had not before existed, was now a troublesome symptom. The respirations were accelerated and labored. Stupor again occurred, with great prostration, and frequent pulse, the respiration continuing accelerated and labored. Diarrhœa, with mucous and bloody stools, occurred. The mouth became aphthous. She had frequent paroxysms of distress accompanied by coughing. In these paroxysms she continued to throw herself about, and cry until exhausted. After this she gradually improved, consciousness returned, the mouth became well, and January 4 she seemed convalescing. The constantly lay on the right side. The pulse continued accelerated, being 130. She was free from pain, had good appetite, and there were no pulmonary symptoms to attract attention. January 22, the parents first discovered enlargement of the chest on the right side. The chest on this side was found to be immovable in respiration; the intercostal spaces elevated; flat on percussion, with no respiratory sound.

Death took place four days afterward.

At the autopsy, twenty-one hours after death, the right chest was found to be filled with laudable, inodorous pus. The lung was compressed into a mass of the size of the hand against the mediestinum. No tubercle. The pleural surfaces covered with layers of fibrin in different stages of organization. Lung on left side free from disease. Heart normal.

Case 3. The patient was a female, twelve years of age. I have already referred to some of the circumstances of the history of this case, under the head of the causation of Chronic Pleurisy. She came under my observation July 16, 1845. She had been attacked suddenly three weeks before with

*pain in the back*, extending from the spine to the lateral portion of the right chest. A swelling on the right side posteriorly and laterally was observed. Dr. Josiah Trowbridge, of this city, saw her twice, and Dr. A. S. Spragu once. Both thought she was laboring under phlegmonous inflammation exterior to the walls of the chest, and looked for the formation of matter in a few days. Poultices were applied until the abscess should be ready to open. Dr. Trowbridge saw her, the first time, a week after the date of attack, and, the second time, a week after the first visit. She was seen by Dr. Sprague two or three days after the last visit by Dr. T. The swelling was tender to the touch, and Dr. Trowbridge thought he discovered fluctuation.

I saw her three weeks after the date of the attack. She was then obliged to maintain a sitting posture. The respirations were accelerated. She complained of pain in the right side. There was manifest enlargement of this side. The intercostal spaces were elevated. Tenderness existed over the right chest posteriorly and laterally. There was universal flatness on percussion over the right side. No respiratory sound save at the summit, before and behind, and in these situations bronchial.

July 19. Made farther record of symptoms as follows: — Has not had any cough, and has none now. No chills or rigors. Had much febrile movement at first. Skin now dry and temperature raised. Pulse 135. Occasionally slight perspiration. Tongue not dry nor coated. Some appetite. Not much thirst. Is more comfortable than on the 16th. Is able to lie down with more ease. She is greatly prostrated; speaks in a very feeble, almost inaudible voice, and the least exertion occasions panting. Intercostal spaces protruded to a level with the ribs, but, on measurement, both sides now equal. Other physical signs the same.

July 17. Much improved. Breathes comfortably, except after exertion. Pulse 100. The feet, since the date of the preceding record, have been very œdematous, but are now of natural size. *Dullness on percussion extends to left side beyond the sternum.* Right side almost immovable in respiration.

August 22. Tumor about three inches below the inferior angle of right scapula, evidently fluctuating. Dr. Trowbridge, who visited the patient with me on this date, stated that the former tumefaction occupied the same site, and that he was satisfied at his former visits that there was matter deeply seated.

August 24. Tumor opened by Prof. Hamilton, and several ounces of laudable, inodorous pus discharged. Flow of pus increased by coughing, and changes of position.

August 29. Free discharge of pus continues, quantity escaping in twenty four hours, estimated at a pint. Expelled freely, and with force, by acts of coughing. General health improved. Appetite good. She is dressed and about the house. Pulse frequent.

September 14. Aspect improved. Appetite good. Has gained in weight and strength. Discharge diminished about one-half. Still laudable pus.

October 27. Much improved. Appetite good. Gained in weight and strength. Able to be out of doors, and to take exercise freely, but is put out of breath more readily than in health. No pain. Discharge continues, varying in quantity on different days. Sometimes a tablespoonful, and sometimes more in quantity. Still healthy pus. Little or no tenderness on pressure. No cough, and at no time has this symptom been present. Full inspiration occasions no pain. Is able to sing with ease and strength. Pulse accelerated. Tongue clean. Right chest contracted, and flat over lower half on all sides. Percussion sound relatively dull above posteriorly. Tympanitic resonance at the upper third anteriorly. Absence of respiratory sound over the portions of chest flat on percussion. Tubular in interscapular space, and obscurely so at the upper part anteriorly. Bronchophony at summit before and behind, and also vocal fremitus.

She has taken no medicine for the last two months. She was bled by Dr. Trowbridge. After my visit she took mercury in combination with squill and digitalis, not carried to ptyalism. The chest was vesicated.

November 16. Still improving.

December 13. Discharge ceased a fortnight ago. Progressively improving in strength and general aspect. Tympanitic resonance at the summit of the right chest anteriorly continues.

November 12, 1847. Patient has not been seen by me since the foregoing date, viz., Dec. 13, 1845, until the present time. She went to school during all last winter, and kept about during the past summer. Last spring pus began to discharge at a point anterior to the old orifice, and also at the latter point. The discharge continued through the summer until two or three weeks ago. Quantity discharged during the twenty-four hours, varied from a tablespoonful to half a pint. She has not been so well since the discharge ceased. She is still able to walk about, but is feeble and emaciated. Easily put out of breath on exertion. Anorexia. Troubled with diarrhœa. Slight cough. Pulse very frequent.

On the right side there is uniform flatness on percussion. No respiratory sound anteriorly or laterally; a faint tubular sound posteriorly at summit. On the left side flatness anteriorly except over an area from two to three inches in diameter at the upper part of the chest toward acromial extremity of the clavicle. Respiratory murmur appreciable in this situation, and over no other part of the chest anteriorly. Percussion yields clear resonance over the left side laterally and posteriorly, and in these situations there is a loud puerile vesicular murmur. Sounds of heart loud. No point of impulse perceptible. Soft bellows murmur with first sound. In respiration the right side is almost quiescent, and on a front view the left shoulder is the only part in active motion.

The coexistence of pericarditis was suspected from the above signs and symptoms.

The patient was not seen afterward, nor the issue ascertained.

It is evident that the causes determining the formation of pus, so far as these few observations go, in pleurisy, are incident to the inflammation; that is to say, when the pleural cavity contains pus, instead of the turbid serum which is usually found, it is not owing to a longer duration of the disease, to a larger amount of effusion, or other circumstances by which it might be supposed ordinary pleurisy is so modified as to run into empyema. Empyema, in other words, would seem to be a form of the disease differing from ordinary pleurisy, *ab initio*, in the tendency to the formation of pus. In each of the three cases just given, pus was formed after the disease had existed but a short time; and, in several of the fatal cases in this collection, in which the disease ended fatally after a much longer duration, and in which the quantity of liquid effusion was equally great, the contents of the chest were found to consist only of serum and fibrin. A larger collection of observations might show that ordinary pleurisy is apt to be converted into empyema; but, in view of the facts contained in this collection of cases, the latter form of the affection involves an intrinsic difference so far as relates to the distinctive feature viz., the formation of pus.*

### MORBID APPEARANCES AFTER DEATH.

The histories in the collection of cases under analysis, embrace eleven autopsies. Aside from those of which an account has been already introduced

* This accords with the view presented by Mr. Paget, in his admirable lectures on inflammation, delivered before the Royal College of Surgeons, of England, (which have fallen under perusal since this Report was written,) of the distinction between "adhesive inflammation," and "suppurative inflammation." Mr. Paget's explanation of the distinction is, that in the former, i. e. in adhesive inflammation, there is a preponderance of the fibrinous element in the effused inflammatory products; while in the latter, i. e. suppurative inflammation, the exudation-corpuscles are in excess.

into the Report, the morbid appearances were such as belong to ordinary Chronic Pleurisy with a greater or less amount of effusion of fibrin and serum, a corresponding compression of the lung, etc. The facts falling under this head are not considered to possess sufficient novelty to warrant a detailed description in the cases individually, which would require considerable space. They are accordingly dismissed with this brief reference.

### TREATMENT.

The few remarks which I shall offer under this head, will be confined to the treatment of simple Chronic Pleurisy, i. e. general pleurisy, not involving perforation, or complicated with other affections. In many respects, however, the same principles of treatment are equally applicable under the latter circumstances. The number of cases in this collection in which the management was observed throughout, is too few, and the plan of treatment too little varied, to institute comparisons in order to determine the influence of different therapeutic measures by numerical results. Nevertheless, on an examination of the treatment pursued, (of which an abstract, as contained in the history of each case, is before me) I may be able to draw some practical conclusions. In doing this I shall avoid, as much as possible, details which the reader would find tedious.

*Bleeding.* General bleeding was practiced in six cases. In all but two of these cases, this remedy was employed before the patients came under my observation. The immediate effects, therefore, were not subjects of study for me, except in these two instances. In one of these the symptoms seemed to be relieved by bleeding; in the other, no alleviation ensued. In the former instance, the case ended in a slow recovery. The latter terminated fatally in seven weeks. In both cases bleeding was practiced twice, shortly after the date of the attack. Of the four remaining cases, recovery followed in two; in one, the patient was able to return to active duties in a month, the chest being, however, still moderately full of liquid effusion; in the other, the recovery was tedious. In the first of these two cases the patient was twice bled. In one of the remaining cases death occurred after five months. This patient was twice bled. The other case was that of the girl who had empyema, which discharged externally. The case, without doubt, ended fatally soon after she was last seen by me, which was more than two years after the date of the attack.

In all the cases in which bleeding was employed, the symptoms denoted acuteness of inflammation, or an approach thereto. The propriety of this measure relates to cases of that description. Few practitioners, if any, at the present time, it is presumed, resort to bleeding in cases in which the disease is subacute at the commencement, excepting, perhaps, some instances in which manifest plethora exists. So far as any inferences are allowable from so few data, the facts which have been cited do not exemplify, in a striking degree, the efficacy of bleeding even in the cases in which the symptoms denote, at the commencement, acuteness of inflammation. The observations, however, are not sufficient in number to authorize any very decided conclusions respecting the value of this remedial measure in this disease.

Local bleeding, by leeching, was employed in two cases, and wet cupping in one. In each of these cases general bleeding was also practiced. Of the two patients leeched, one died, and the other recovered. The patient cupped, recovered. These facts are not mentioned because they are supposed to have much, if any value. They are obviously inadequate for any practical inferences.

*Mercurials and Diuretics.* I include these remedies in the same category because they were almost uniformly given in conjunction. Simple mercurialization, without diuretics, or other remedies, was not tried in any instance. In general, the plan of medication pursued was that advised by Dr. Hope, consisting of mercury, with diuretics, or cathartics, followed by, or associated with tonics, nutritious diet and vesication over the chest. With respect to the special agency of mercury, therefore, my observations do not furnish any definite results. The same remark will apply, measurably, to other remedies; but not to the same extent, because diuretics, and in some instances cathartics, etc., were continued after mercurials had been suspended. The object kept in view in directing these measures was chiefly the absorption of the liquid effusion. In several instances the effect of mercury and diuretics in this respect was marked. If the quantity of urine was increased in any considerable degree, the accumulation within the chest was sensibly diminished. This diminution, however, proceeded only to a certain extent. The liquid contents were in no instance speedily and entirely absorbed; but sufficient effect was produced to cause decided relief to the symptoms due to distension, and also to lead to an obvious contraction of the dimensions of the chest. Mercury was never carried beyond slight ptyalism, and not uniformly to that point. The diuretics used were squill, digitalis, the supertartrate, and the nitrate of potash. These were generally given more or less conjointly, or rather simultaneously. A dry diet was usually enjoined, especially while diuretic remedies were prescribed.

*Cathartics.* In some cases it was observed that the kidneys were no readily acted on to produce increased secretion; and, occasionally, watery evacuations from the bowels took place under the use of diuretic remedies. Under these circumstances, hydragogue cathartics were employed. The liquid effusion was sometimes apparently diminished by their operation. Gamboge, elaterium, the sulphate of magnesia, and, in one case, podophyllum were prescribed for this object.

*Iodine.* This remedy was given in a few cases, but without marked effect in promoting absorption of the liquid effusion.

*External applications.* These consisted of vesication, stimulating liniments, and the iodine ointment. Vesication was uniformly employed, to a greater or less extent, after the mode advised by Dr. Hope, viz., small blisters, removed early, and carried successively over different parts of the affected side. The impression derived from the constant use of this remedy was not unfavorable to their efficiency in favoring the process of absorption. It is true that the blisters were usually conjoined with other therapeutic measures, but in some instances they were continued with advantage after other remedies had been suspended. Stimulating liniments were employed simply to relieve pain. The iodine ointment was resorted to in a few instances without any special effect being observed.

*Tonics, stimulants, diet, and regimen.* After the use of the measures already referred to had been continued for some time, and sometimes before they were discontinued, tonic remedies, and diffusible stimulants generally constituted the medicinal treatment. They seemed to exert a good effect, and in some instances this was striking. Conjoined with their use, a generous diet, and moderate exercise in the open air were found highly useful. In several cases it appears from the histories that the patients after a certain measure of improvement, remained in a stationary condition until they began to go out of doors. In one case the patient had for several weeks been confined to the bed, and ulceration of the nates occurred in consequence. Under these circumstances, he was carried out, placed in an easy carriage, and rode a short distance. He returned much improved. The plan was systematically continued, with constant improvement, and from that date he steadily and rapidly advanced in his recovery. In a less striking degree the salutary influence of the same regimen was equally obvious in several instances.

In thus endeavoring briefly to state the results of my experience in the management of Chronic Pleurisy, the want of precision is not less obvious to the writer, than to the reader. Exactness in determining the relative agency of different measures of treatment is, however, seldom attainable; and especially when the treatment consists of several measures employed in conjunction, or succession, the difficulty of ascertaining experimentally the influence which belongs to them separately is very great. Moreover, with respect to the disease under consideration, as also many other diseases, science has not yet acquired the true standard by which to measure the efficiency of remedies individually or collectively. This standard is the natural history of disease uninfluenced by medicinal management. A series of cases in which Chronic Pleurisy was allowed to run its course, under favorable hygienic conditions, without remedies, would furnish results of very great value as a point of departure for determining how far therapeutic agencies are really efficacious. The difficulties in the way of obtaining such a collection of facts, it is obvious, are of a nature to render it doubtful if they will ever become the property of science. As an approximation to an end so desirable, the amount of fatality under different modes of treatment is to be considered. The proportion of deaths, in the present collection, among the cases of simple uncomplicated pleurisy has been seen to be quite small. In the collection of seventy-eight cases analyzed by Dr. Blakiston, to which reference has before been made, recovery took place in every instance. Dr. B. quotes a statement by Louis, made to the Academy of Medicine at Paris, "that he had never met with a case that had terminated fatally when the disease was in its simple form." That the vast majority of cases end in recovery, certainly accords with the general experience of medical practitioners. It is, therefore, fair to presume that the natural tendency of the disease is toward a favorable termination. This consideration is to be taken into account in estimating the importance of remedies severally, or combined. When the disease proves fatal, it is by being complicated with other affections, which are but little under the control of art.

To sum up, in a few words, the practical views of the management of Chronic Pleurisy based on the personal experience of the writer, the first and chief object in the early part of the disease, is to effect absorption of the liquid effusion. It is an object, doubtless, to prevent or limit the effusion, if cases come under observation, and the diagnosis is made before this takes place to much extent. But in a large proportion of instances the effusion takes place so rapidly, and is accompanied by so little pain or other symptoms of prominence, that at the first examination the chest is found to be already filled. Mercury and diuretics exert a certain amount of efficiency in causing absorption to commence, and continue. If the kidneys are not readily acted on, hydragogue cathartics may exert a similar influence. Small blisters applied in succession, in a manner least liable to occasion constitutional irritation, are valuable means for the same end. The effect of iodine applied locally, and administered internally, is less appreciable, but perhaps not altogether negative.

The beneficial results of these therapeutic measures have certain limits, both as respects the extent to which they promote absorption, and the duration of their employment. The complete removal of the liquid effusion by absorption, requires usually a long period, usually several months at least. The remedies mentioned should not be persisted in after they cease to exert an obvious effect. Fortunately the resources of physical exploration, in this disease, enable us to determine accurately, from day to day, the condition of the chest. If the accumulation within the cavity of the pleura does not sensibly diminish, but remains stationary for days or weeks, it is useless to continue measures which may do positive harm by compromising the powers of the constitution. Considering the natural tendency of the disease toward final recovery, or at all events the absence of a tendency to a fatal termination, we should not be justified in subjecting the system to the long continued operation of medicinal agencies which must of necessity prove hurtful, if they fail to accomplish the desired end.

After giving the measures just referred to a fair trial, reliance must be had on tonic remedies, diffusible stimulants, associated with a generous diet, and an invigorating regimen. To preserve the powers of the constitution in an affection requiring so long a period for the completion of recovery, is a prominent end to be kept in view in the management. My observations have furnished repeated illustrations of immediate and progressive improvement directly all debilitating medicines were suspended, and a sustaining plan adopted. So far as my experience goes, the effect of this change in the treatment has been more striking than the apparent efficiency of any medicinal remedies. The duty of guarding and developing the vital forces, in my estimation, ranks first in importance. It is almost needless to add, that due protection against exposure, over-exertion, etc., is by no means to be overlooked. Exercise in the open air should be restrained within proper limits, the surface of the body is to be kept at an uniform temperature by appropriate clothing, and dietetics duly regulated. The patient should not go out when the weather is inclement. Flannel, or raw silk, should be worn next the skin, and in winter vestments of buckskin or chamois leather may be superadded. The food should be substantial and plain, with a fair proportion of flesh, and a sparing supply of liquids. Excesses and irregularities of all kinds are of course to be interdicted.

In conclusion, a few remarks on the subject of *paracentesis thoracis* may not be out of place in this Report, although my observations contain no facts relative to this operation. This subject has of late given rise to considerable discussion, and in this country the attention of the profession has been directed to the importance of the operation by Dr. H. I. Bowditch,* of Boston, Mass., who has reported several cases in which it was practiced. One object in Dr. Bowditch's communications has been to bring to the notice of practitioners a new method of performing the operation, devised by Dr. Morrill Wyman, of Cambridge, Mass. This method consists in using an exploring canula and trochar, which is attached by a flexible tube to a suction pump so constructed that the fluid may be removed from the chest through the canula, and discharged from the pump through another aperture.

Dr. Bowditch gives a report of eight cases in which this method was resorted to. Three of the cases ended in recovery, and the operation is considered by Dr. B. to have had an important influence in the favorable result. In three cases material improvement followed the operation as respects the rational and physical signs. In no instance were there any unpleasant consequences aside from the slight inconvenience from the punctures. The operation is shown to be made with ease, and apparently with entire impunity.

As regards the results, Dr. Bowditch was unfortunate in the selection of cases in which trial was made of this plan of treatment, inasmuch as the proportion of recoveries (3–8) is much less than would be expected to occur in cases coming indiscriminately under observation, and managed without resorting to paracentesis. It is probable that Dr. B. did not feel justified in making trial of the operation except in cases in which the symptoms were unusually severe. This is the impression received on reading the details of the histories; and it does not appear, as stated by Dr. B., that in any case the symptoms or progress of the disease were unfavorably affected by giving exit to a portion of the liquid contents of the chest. Moreover, several of the cases reported by Dr. B. were cases of empyema, and it is well known that, the absorption of pus being effected with much greater difficulty than when the liquid effusion consists of fibrin and serum, this variety of Chronic Pleurisy is much more likely to prove fatal than the simple form.

Rationally considered, the operation, performed after the mode suggested by Dr. Wyman, is an important improvement in the management of Chronic Pleurisy, and appears to be free from any serious objections. The end of medication is to effect removal of the liquid effusion, by means of absorption. If this end can be attained by a simple, trivial operation, devoid of all danger,

*Am. Med. Jour. of Med. Sciences, No. for April, 1852.

it would certainly seem that an advantage has been gained, inasmuch as the agency of remedies for the same end is uncertain, slow, often ineffectual, and exposes the patient to injury. If it be found by experience to be hurtful to remove the contents of the chest at once, or in a short space of time, and that, therefore, the gradual operation of the process of absorption is preferable, this difficulty may be easily avoided by repeating the operation at such intervals as observations show to be most judicious, and removing a small quantity at a time. The operation is so slight, and attended with so little inconvenience, that there is no objection to this course. In cases in which the accumulation is large, producing great distress, and perhaps threatening life, and especially when other remedies fail to procure relief, there can be no question as to the propriety and importance of paracentesis, even performed after the old method. In cases of empyema, also, it cannot be doubted that it is desirable to withdraw the purulent liquid artificially, and not trust to absorption. The difficulty here consists in determining the fact of the chest containing pus. As already remarked under another head, where this form of pleurisy is suspected, there seems no objection to the use of the exploring canula as an instrument of diagnosis.

Heretofore, the question of the propriety of resorting to paracentesis involved the liability of mistaking for pleurisy with effusion, other affections of the chest. The operation has frequently been made when the chest contained no liquid. At the present time, however, uncertainty in diagnosis is not a valid reason for omitting or delaying to puncture the chest. Physical exploration supplies demonstrative evidence of the presence of liquid in the cavity of the pleura. It remains by accumulated observations to determine whether in simple Chronic Pleurisy with effusion, the operation of paracentesis may be resorted to without diminishing the chances of recovery, and with the effect of relieving unpleasant symptoms, and expediting recovery.

